# Timed picture naming norms for 800 photographs of 200 objects in English

**DOI:** 10.3758/s13428-024-02380-w

**Published:** 2024-03-19

**Authors:** Rens van Hoef, Dermot Lynott, Louise Connell

**Affiliations:** 1 Department of Psychology, Fylde College, Lancaster University, Bailrigg, Lancaster, LA1 4YF UK; 2 Department of Psychology, Maynooth University, Maynooth, Co. Kildare Ireland

**Keywords:** Picture naming, Object recognition, Language production, Naming uncertainty, Response time

## Abstract

The present study presents picture-naming norms for a large set of 800 high-quality photographs of 200 natural objects and artefacts spanning a range of categories, with four unique images per object. Participants were asked to provide a single, most appropriate name for each image seen. We report recognition latencies for each image, and several normed variables for the provided names: agreement, *H-*statistic (i.e. level of naming uncertainty), Zipf word frequency and word length. Rather than simply focusing on a single name per image (i.e. the modal or most common name), analysis of recognition latencies showed that it is important to consider the diversity of labels that participants may ascribe to each pictured object. The norms therefore provide a list of candidate labels per image with weighted measures of word length and frequency per image that incorporate all provided names, as well as modal measures based on the most common name only.

## Introduction

Pictures and photographs of objects are widely used as stimuli in many fields of research, such as perception, memory, cognition, and language processing. However, pictures may vary on a wide range of characteristics, such as visual (e.g. colour, texture), semantic (e.g. concept familiarity) as well as the lexical characteristics of the labels they elicit (e.g. word frequency, name agreement; see Alario et al., 2004; Perret & Bonin, 2019 for reviews). To allow experimental control of the variability associated with an image, researchers have created standardised sets of pictures, which describe their visual and semantic characteristics, as well as the lexical characteristics of their associated names. Normed picture sets have been used in psycholinguistic (Ostarek & Vigliocco, 2016; Vinson et al., 2015), object recognition (Bramão et al., 2011; Catling et al., 2008; Rossion & Pourtois, 2004) and neuroimaging research (Gerlach, 2009; Thompson-Schill et al., 1997).

### Colour photographs vs. line drawings of objects

To date, most picture-naming norms comprise line drawings. Arguably, the most influential set of picture-naming norms is the one compiled by Snodgrass and Vanderwart (1980). This set consists of a standardised database of 260 black and white line drawings depicting natural objects and artefacts from a range of categories, and their associated values for image agreement, familiarity, and complexity as well as name agreement for their given names in English. Since their creation, the Snodgrass and Vanderwart norms have been extended by other researchers, who have included more images and/or collected their names across multiple languages (e.g. Bates et al., 2003; Sanfeliu & Fernandez, 1996; Severens et al., 2005), added colour to drawings (Rossion & Pourtois, 2004), investigated further psycholinguistic variables (e.g. age of acquisition: Barry et al., 1997) and added naming times (Snodgrass & Yuditsky, 1996). Moreover, the Snodgrass and Vanderwart norms have remained relevant as the blueprint for a range of other normative studies using line drawings (Bonin et al., 2003; Duñabeitia et al., 2018, 2022; Martínez et al., 2020). The pervasiveness of line drawings rests on the assumption that they are processed similarly to more realistic depictions (Salmon et al., 2014), but are easier to produce and control (e.g. an artist may draw a wide range of objects in any orientation). However, as Brodie et al. (1991) note, the adequacy of this approach is rarely addressed in norming studies.

Photographic stimuli are often preferred to line drawings, primarily because of their greater ecological validity and because they lend themselves more easily to experimental manipulations of physical properties such as colour, luminance, or spatial frequencies (Moreno-Martínez & Montoro, 2012; Navarrete et al., 2019; Viggiano et al., 2004). Indeed, colour photographs tend to result in better object recognition than either black-and-white or colourised line drawings (Bramão et al., 2010; Heuer, 2016; Price & Humphreys, 1989; Salmon et al., 2014; see also Sanocki et al., 1998). Such an advantage for photographs is consistent with simulated (or grounded, embodied) accounts of conceptual processing, which hold that concepts are represented in semantic memory at least in part as partial simulations of sensorimotor, affective and other experience with our environment (Barsalou, 1999; Meteyard et al., 2012). For instance, grayscale photographs of manipulable objects are named faster than black-and-white line drawings of the same objects (Salmon et al., 2014), supporting the idea that realistic object depictions in photographs facilitate greater activation of relevant motor areas than do line drawings.

One potential criticism of full-colour photographs compared to line drawings is that a photograph depicts an individual instance or *token* of the object whereas outline drawings may instead depict a generic class or *type* (Uttl et al., 2006). For this reason, Heuer (2016) warns that the visual cues in photographs may make them more susceptible to viewer bias and argues that they primarily facilitate object recognition for populations that are familiar with the depicted instance (e.g. a bar of soap from a UK brand facilitating recognition in a UK population). However, familiarity-based viewer bias is not exclusive to photographic material. For example, the Snodgrass and Vanderwart (1980) picture set contains a depiction of a standard North American electrical plug (image 178), which may be unfamiliar to British, mainland European, or Australian populations (each of which uses a different plug). Similarly, its image of a roller skate (image 189) deviates considerably from current skate forms that modern-day populations might find familiar. Experimenters should strive to ensure that the stimuli they use are representative and familiar to the population they aim to test, regardless of whether they intend to use photographs or line drawings. An alternative approach for photographic norms is therefore to provide multiple images of each object (i.e. multiple tokens per type) to allow experimenters to select object instances that are likely to be familiar to their test populations. Such an approach to picture norming also allows experimental designs to decouple object repetition from image repetition, such as when pairing a pictured object with multiple cues (e.g. van Hoef et al., 2023) or when presenting objects in separate learning and test phases (e.g. Dymarska et al., 2022). The present picture-naming norms thus collect English names for four photographs each of 200 distinct objects (i.e. 800 images in total).

### Characteristics of object names

As well as the pictures themselves, picture-naming norms also of course include the names of the depicted objects and, more recently, the latencies for producing these names. However, the names that participants spontaneously ascribe to pictured objects can vary enormously (e.g. a picture of a sofa might be named as a *sofa*, *couch*, *seat*, or in some dialects *settee*, and so on) which in turn affects latencies. Indeed, previous work has found strong evidence for the effects of naming uncertainty per image (as expressed by the *H-*statistic; Lachman, 1973) and name agreement or codability per image (i.e. how consistently or easily do participants give the same name to an object) on response times in picture-naming and recognition (e.g. Székely et al., 2003). Normed name agreement, in particular, can predict both the frequency distribution of picture names in new samples of participants (i.e. within-population variation) and the likelihood of an individual activating a name on a given occasion (i.e. within-participant variation), although the latter is also subject to individual preferences (Balatsou et al., 2022).

Other variables typically included in picture-naming norms relate to characteristics of the modal (i.e. most commonly given) name per image, such as word frequency (written, spoken) and word length. However, evidence for the effect of these variables on naming latencies is not consistent. For example, while researchers previously found evidence for the effects of written (Snodgrass & Yuditsky, 1996) and spoken word frequency (Ellis & Morrison, 1998) and of word length (D’Amico et al., 2001; Székely et al., 2003) on response latencies in object naming, a recent meta-study (Perret & Bonin, 2019) of 18 normative studies containing black-and-white line drawings found inconclusive evidence for the effects of word frequency and length. One possible reason for the lack of a clear word frequency effect on naming is the fact that the methods used to collect word frequency vary considerably across different studies. Brysbaert et al. (2018; see also Johns & Jamieson, 2019) suggest that corpus selection tailored to the language to which participants are most frequency exposed allows for better prediction. For example, of the studies used in Perret and Bonin (2019), five tested undergraduate populations using frequency ratings based on written texts (e.g. novels, essays, poems, dramatic works, non-fiction books, newspaper articles and magazines), which were collected years (e.g. Nishimoto et al., 2005; Pind & Tryggvadóttir, 2002) and sometimes even multiple decades (e.g. Bonin et al., 2002; Perret & Laganaro, 2013) prior to testing, and hence may not have reflected the language their participants were exposed to. In the present norms, where we collected naming data from native speakers of English in the UK, we therefore used word frequencies obtained from a large corpus of program and film subtitles from contemporary British television (van Heuven et al., 2014).

Another possibility for the lack of robust word-level effects is that restricting analyses to the word frequency of only the *modal* name is too narrow a source of information to capture the intended effect. For instance, in some cases the modal name ascribed to an object is produced by a relatively small proportion of participants, such as *beetle* (50% agreement; competing names include *insect, bug, cockroach*: Snodgrass & Vanderwart, 1980) or *antelope* (39% agreement: competing names include *springbok, deer, gazelle*: Adlington et al., 2009). Since the effects of name agreement and uncertainty indicate that the number of competing names strongly affects performance, it is possible that including the frequency and length of these competing names may bolster the predictive power of word frequency and length. Therefore, the present study also includes the *weighted* average word frequency and length per image, where these variables are weighted by the proportion of participants who produce each name for a given image.

In summary, the present study aimed to create a large set of photographic picture-naming norms for objects across a range of artefactual and natural categories, featuring multiple high-resolution photographs per object (see OSF for norms, images, and attribution: https://osf.io/r3hbz/). In the present norms, we report the *H-*statistic (measure of uncertainty in labelling an object; Lachman, 1973), name agreement (percentage of participants that gave the modal name), modal-name word length in characters, and modal-name Zipf log word frequency for every image in our set. These variables are consistent with previous picture-naming norms, are linked to the name and response activation stages of picture-naming as outlined by Johnson et al., (1996) and Alario et al. (2004), and form the basis of our comparison with previous norms (i.e. convergent validity). Furthermore, we report mean naming latencies per image and explore whether the weighted word frequency and length of *all* responses given to an image explain response latencies better than word frequency and length of the modal name only.

## Picture-naming norming method

### Participants

Sixty participants (31 female; M_age_ = 37.23 years, *SD* = 13.00) were recruited through online recruiting platform Prolific (www.prolific.co). Using Prolific’s custom pre-screening, we selected participants to be native speakers of English with British nationality, have normal or corrected-to-normal vision, and no reading impairments (e.g. dyslexia). Participants were paid per completed study section (see procedure), starting at £2.50 for completing the first section (consisting of four image lists) and £0.50 for every additional section (consisting of one list each). Participants could submit between 4 and 16 complete lists as desired. On average, participants completed 8.62 lists (*SD* = 3.99), which – at 50 images per list – meant that each participant provided an average of 430.83 picture-naming responses (*SD* = 199.60). The supplementary materials on OSF contain a detailed breakdown of the number of responses per participant, list, and image.

We validated our sample size by testing for the average modal name agreement, *H*-statistic as well as the average proportion of all responses that were idiosyncratic, following the coding scheme outlined in O’Sullivan et al. (2012). Where the former two statistics are common measures of name agreement, the percentage of idiosyncratic responses for an image captures additional information about the number of participants that recognised but did not use a typical name for the image (see response coding below). At 60 participants, means of all three measures in our norms fell between the values reported by O’Sullivan et al. for the Snodgrass and Vanderwart (1980) and BOSS (Brodeur et al., 2010) picture-naming datasets, suggesting our norms feature typical patterns of consistency in picture-naming behaviour: modal name agreement = 66.25% (*SD* = 22.02%), *H-*statistic = 1.38 (*SD* = 0.82), proportion of idiosyncratic responses per image (*M* = 2.27%, *SD* = 4.51). Data collection was approved by the Lancaster University Faculty of Science and Technology Research Ethics Committee. All participants read information detailing the purpose of and expectations of the study and gave informed consent which included the acknowledgement they would be paid for each completed (but not partially completed) section of the study and explicit permission to share all anonymised alphanumeric data publicly.

### Materials

The stimulus set consisted of 800 full colour images of 200 objects (100 natural objects, 100 artefacts; see supplementals on OSF for the full set of photographs), which depicted only the target object on a white background. The 100 natural objects belonged to 23 basic-level categories (e.g. dog, cat, bird, lizard, fish, insect, tree, vegetable, fruit etc.). The 100 artefact objects belonged to 26 basic-level categories (e.g. boat, box, car, cup, aircraft, watercraft, snowcraft, case, bag, ball, truck, tool, etc.).

For each category, we collated a set of candidate member objects (e.g. types of dogs) through various means such as category production norms (e.g. Banks & Connell, 2023), WordNet (Miller, 1995), and by free generation. As we intended these norms to be compatible with Lynott et al.’s (2020) norms of sensorimotor strength, we selected objects whose presumed name was present in the Lynott et al. norms. For each candidate object, we attempted to source four photographs through Google image search. We selected photographs to be free for use with modification (please see supplemental materials for attribution information for all images), to depict the target object clearly and without obstructions that rendered it unidentifiable, and to have a minimum size of 1024 × 768 pixels. Where we could not find four suitable images, we removed the object from the set of candidates. In order to ensure a variety of objects across the norms, we allowed no more than ten members per category (e.g. when we had suitable images for ten types of *dog*, we moved onto sourcing images of different kinds of *boat*).

**Fig. 1:**
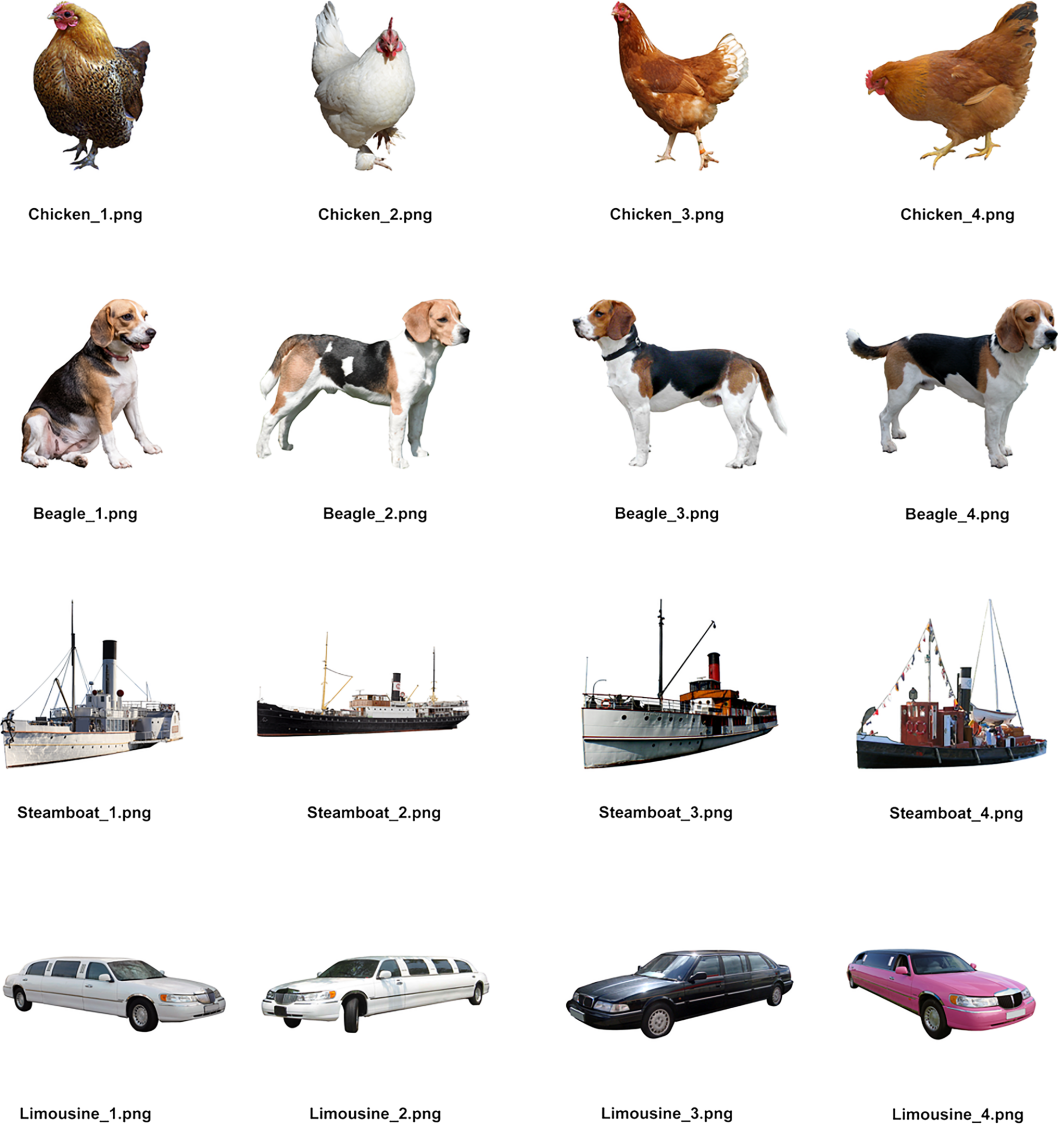
Examples of all four images for various objects

We edited all photographs with Adobe Photoshop 2020 (version 21.2.3). Specifically, we cut each target object from the original photograph and placed it centrally on a white background sized 1920 × 1080 px, with a minimal margin of 200 pixels on every side (see Fig. 1). In addition to this, we removed any visible text and distracting objects (e.g. we removed humans from a *steamboat* image).

### Procedure

We designed and hosted the experiment on Gorilla.sc (Anwyl-Irvine et al., 2020). We pseudo-randomly divided all photographs over 16 lists of 50 trials each, such that images of each given object were distributed across at least two different lists. Lists were rotated across participants, where participants were randomly assigned to one of four starting lists (list 1, 5, 9 or 13). If a participant opted to complete more than the first four lists, they were presented with the next list in sequence (e.g. participants who started with lists 1, 2, 3, and 4 subsequently saw list 5, 6, 7 and so on), until a participant decided they did not want to complete more lists, or they had completed all 16 lists.^1^

Participants were instructed that they would be shown a series of photographs, and that each photograph depicted one object. They were asked to press the *spacebar* as soon as a name for the depicted object came to mind, and to enter that exact name in the text box that followed each photograph, or to enter *DK* for ‘don’t know’ if they did not know what an object was. Trials were presented in a randomised order. Each trial was presented centred on a white background in the participant’s browser window, and started with a fixation cross for 200 ms, followed by the photograph, which remained on screen until participants indicated they recognised and could name it by pressing the spacebar. Once a participant pressed the spacebar, the photograph disappeared and was replaced by a textbox in which they could enter a name. We recorded RT from the onset of the photograph until participants pressed the spacebar on their keyboards (object recognition RT) and recorded the object name that participants subsequently entered in a typed naming paradigm (e.g. Torrance et al., 2018).

A brief practice session with four items (*monkey wrench*, *screwdriver*, *skirt,* and *armadillo*), that were not present in the experimental stimuli set nor belonged to the same basic-level category as any of the experimental stimuli, familiarised participants with the experiment procedure. In this practice session, participants received reminder instructions at each step of the trial (e.g. “Press spacebar as soon as a name for this object comes to mind” and “Please enter the first name that came to mind when you saw this object and press enter/return on your keyboard to submit and continue to the next image”). These instructions were absent in the main testing session. At the end of each list in the main testing session, participants were presented with the number of lists they had completed up to that point and had the option to stop or to complete another list. Testing took approximately 15 min for completing the first four lists, including participant information, informed consent and debrief, and took approximately 3 further minutes for each additional list completed. Trial-level response data is available on OSF (https://osf.io/r3hbz/).

### Response coding

**Fig. 2:**
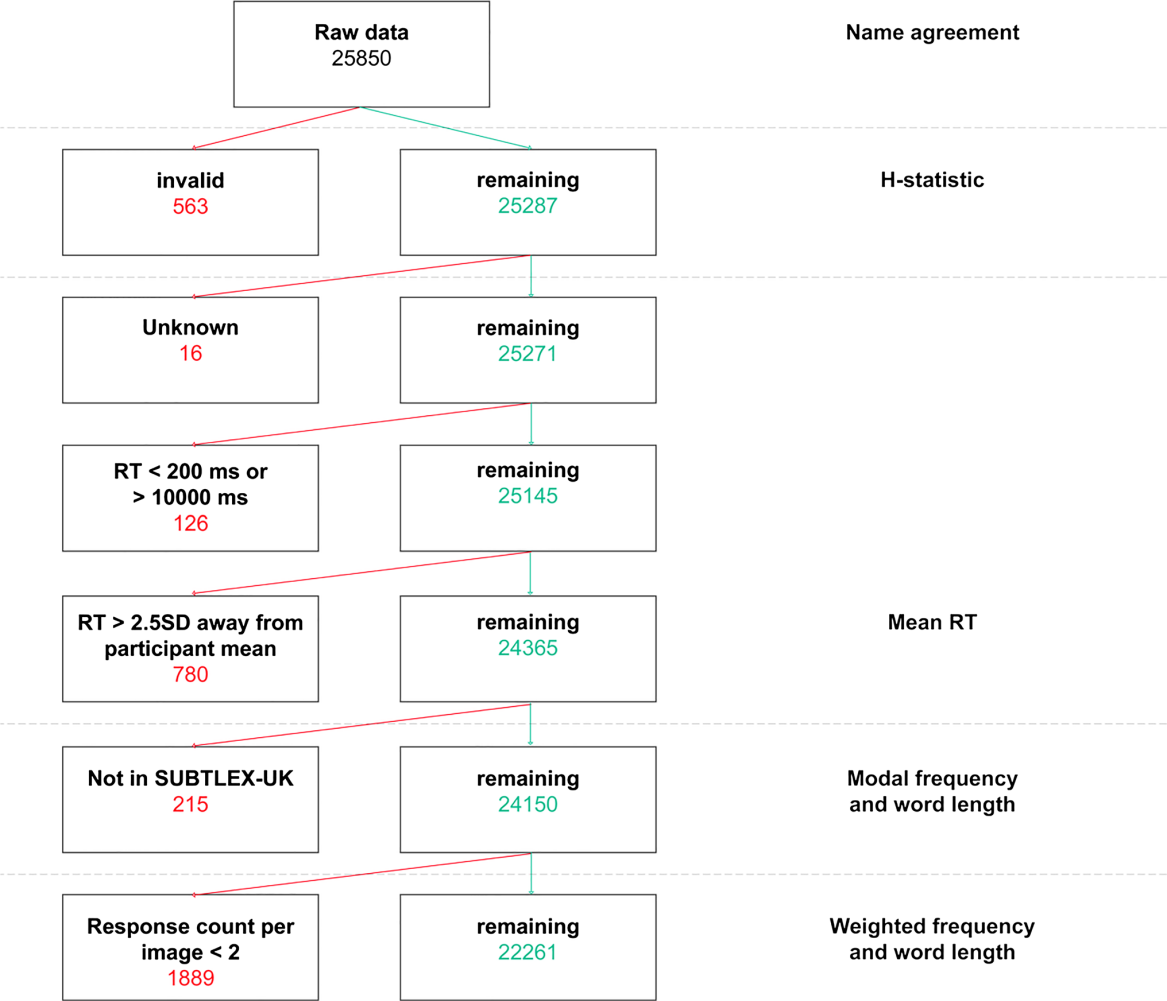
Breakdown of removal of trial-level response data and corresponding image-name combinations and variables calculated from each subset

In line with previous picture-naming norms that used a typed naming paradigm (e.g. Torrance et al., 2018), we first clustered different spellings of the same name, and coded them as instances of a standardised, correctly spelled group name (e.g. for an image of a chihuahua, responses *chiwawa,* *chiuaua* and *chihuahua* were all grouped and coded as instances of the name *chihuahua*). We coded all ‘DK’ or equivalent (e.g. *‘n/a’, ‘picture did not appear’*) as *invalid* (*n* = 563 or 2.18% of all 25,850 responses). These invalid responses were included for the calculation of name agreement (see Normed variables, below) but excluded for the calculation of the *H*-statistic (see Fig. 2).

In line with O’Sullivan et al. (2012), we sought to allow for insight into reasons for name disagreement by coding *equivocal* responses (i.e. *unknown*, *idiosyncratic* and *non-object* responses). We coded as *unknown* all participant responses that were not directly understandable in relationship to the image and do not occur in the English dictionary (*Cambridge Dictionary | English Dictionary, Translations & Thesaurus*, 2023; *n* = 16, .06% of valid responses). This category included blank responses, or strings (partially) consisting of random letters that could not be identified as invalid responses (e.g. “surf paf”, “piole”, and “par”). These unknown responses were excluded from the calculation of average recognition RT. Furthermore, we coded as *idiosyncratic* all responses that were understandable in relationship to the image but do not occur in the English dictionary (*n* = 588, 2.32% of valid responses). These responses consisted predominantly of compound responses (e.g. “road flattening machine”) that were unique to a select number of participants. In addition to this, we coded as *non-object* all responses that were understandable in relation to the image and occur in the English dictionary but did not refer to a single object (*n* = 246, 0.97% of valid responses). This category included verbs describing an activity (e.g. *skiing, flying*), non-object nouns (e.g. *health*), adjectives (e.g. *old*), geographical features (e.g. *sea*, *beach*), and materials (e.g. *gold, wicker, wood*). Equivocal responses were included in calculation of normed variables, with one exception: unknown responses were excluded for the calculation of average RT.

In contrast to previous studies, which sought to validate pre-existing picture sets (e.g. Bates et al., 2003; Rossion & Pourtois, 2004), our study included only images for which (to our knowledge) no previous naming distributions had been determined. As such, we could not discount responses that failed to match a predetermined target answer (e.g. Snodgrass & Vanderwart, 1980). Instead, we adopted a strategy more akin to the lenient correctness scoring variant in Snodgrass and Yuditsky (1996) and included all responses even if they were clearly erroneous (e.g. if more than one person thought a *computer mouse* was a *helmet*, then they may have had a point about what the image resembled). However, we did observe a small number of responses (*n* = 14, 0.05% of valid responses) that could not be assumed to describe the depicted object but did not fall in any of the equivocal categories. Many of these responses seemed to be associative rather than descriptive (e.g. ‘*Sunday roast* for a picture of a *carrot*, *‘sunflower oil*’ for a picture of a *sunflower*). We coded these responses as *physically dissimilar* but did not exclude them from calculation of normed variables (Fig. 2).

### Normed variables

The norms contain the following variables for each image:

#### Names

The most common name produced for each image (i.e. the modal name^2^), as well as all alternative names 1-k (i.e. non-modal names listed in descending order of production frequency).

#### Production frequencies

The number of participants that produced a given name for an image. In the image-level norms, we reported raw production frequency of the modal name per image (modal production frequency), as well as individual raw production frequencies for each non-idiosyncratic non-modal name (alternative name production frequencies).

#### Agreement

The percentage of participants that gave the modal name for every image, based on all responses.

#### H-statistic

The information theoretical *H-*statistic (entropy) for each image, calculated as per Snodgrass and Vanderwart (1980) and Rossion and Pourtois (2004):

$$H= \sum_{i=1}^{k}{p}_{i}{{\text{log}}}_{2}\left(\frac{1}{{p}_{i}}\right)$$

where *k* is the number of valid responses (i.e. excluding invalid responses, see *Response coding*, above) per image, and *p*_i_ is the proportion of participants who produced each response. The *H-*statistic is a measure of uncertainty in labelling an object (Lachman, 1973) and has been frequently shown to be a stronger predictor of naming latencies than simple name agreement (Bates et al., 2003; Severens et al., 2005; Snodgrass & Vanderwart, 1980; Székely et al., 2003; Torrance et al., 2018). Low values of *H* represent low uncertainty amongst participants (i.e. a high level of consensus in naming: *H-*statistic = 0 means all participants gave the same name for an image) whereas high values of *H* represent high uncertainty (i.e. great diversity in naming, where participants produce many different names for a given image). In the norms, we reported one *H-*value per image. As the *H*-statistic is sensitive to variations in sample size (e.g. due to removal of invalid responses), we also reported the normalised *H*-statistic (Krautz & Keuleers, 2022) in order to allow for meaningful comparison between uncertainty for various images.

#### RT

Recognition RT, measured from the onset of the image to the onset of a valid keypress indicating a participant had recognised the object and a name had come to mind. In the norms, we included the overall mean Recognition RT per image, averaged over all valid, known (i.e. not coded as *unknown*, see Response coding), non-outlier responses. Specifically, out of 25,271 valid, known responses, we removed 95 responses with recognition RTs longer than 10 s (reflecting participant inattention) and 31 responses with recognition RTs below 200 ms (motor error). All participants had a mean recognition RT within 2.5 SD of the overall mean, and so no participants were excluded outright on that criterion. Finally, we removed as outliers 780 recognition RTs that were at least 2.5 SD away from the relevant participant’s mean.^3^

#### Word frequencies

Zipf word frequency for all spelling-corrected, valid, non-idiosyncratic, unigram names was derived from a corpus of subtitles in British English (SUBTLEX-UK: van Heuven et al., 2014). The Zipf scale is a logarithmic scale, ranging from approximately 1 to 7, with 1 corresponding to a wordform frequency of 1 per 100 million words (very low frequency: e.g. *antifungal*, *milia*), and 7 corresponding to a wordform frequency of 1 per 100 words (very high frequency: e.g. *and*, *to*). For two-word object names (e.g. *fire engine*), we calculated bigram Zipf frequencies from their raw bigram frequencies provided by van Heuven and colleagues. The average Zipf frequency across all unique responses was 4.03 (*SD* = .91), with Zipf frequencies ranging from 0.997 (e.g. “salad onion”, “snow ski”) to 6.56 (“can”).

In the norms, we included Zipf word frequency for the modal name (modal word frequency), as well as the average Zipf word frequency of all names produced for an image, weighted by their relative production frequency (weighted word frequency). In calculation of weighted word frequencies, we excluded any responses where word frequency was unavailable for the name (83 non-modal names, featured in *n* = 215 responses) as well as responses that occurred only once per image (*n* = 1889 responses).^4^ These latter responses were unique to a single participant and were typically coded as equivocal (e.g. *unknown*, *idiosyncratic* and *non-object* responses: see Response coding) that were thus unlikely to be representative of what names people activate upon seeing an image.

#### Word lengths

We calculated word length as the number of letters in the final spelling-corrected names, excluding spaces (e.g. *palm tree* had a length of eight letters). In the norms, we included word length of the modal name for every image (modal word length), as well as the average name length of all names produced for an image, weighted by their relative production frequency (weighted word length). In addition, we included individual word lengths for each non-idiosyncratic non-modal name (alternative name word lengths).

### Summary of variable characteristics

**Fig. 3:**
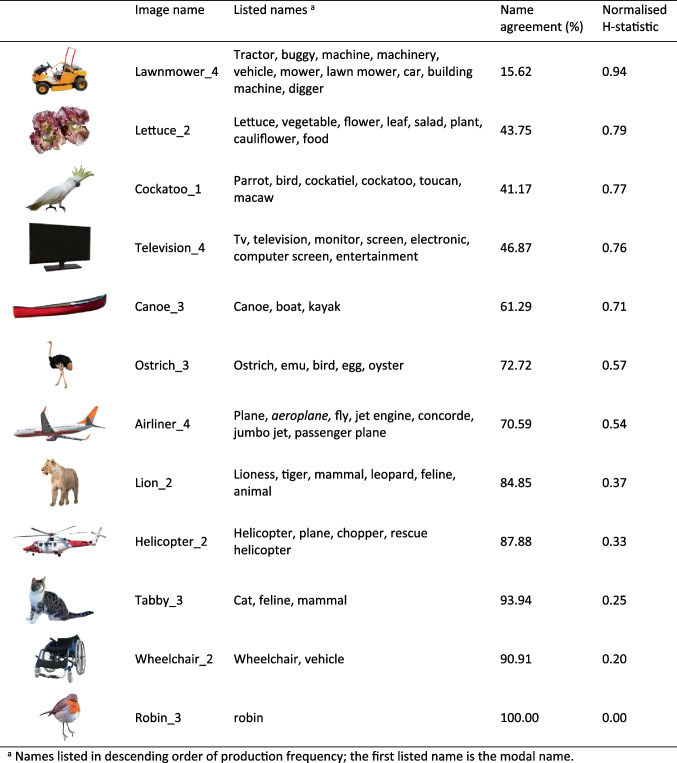
Examples of the listed names for a sample of artefact and natural objects, ranging from low to high name agreement and *H*-statistic

The median number of valid, known responses per image was 32 (min = 13, max = 34). The median number of unique names per image was 5 (min = 1, max = 16). We calculated summary descriptive statistics for all variables, per image and per name (see supplementals). Table 1 shows overall descriptive statistics for each variable (name agreement, *H*-statistics, word length, word frequency and response time). Figure 3 provides a range of example images of natural and artefact objects with high, average, and low naming agreement.

**Table 1:** Image-level summary statistics: average name agreement, *H*-statistic (standard and normalised), recognition RT (in ms), modal production frequency, modal word frequency, modal word length, weighted word frequency, and weighted word length

Variable	Mean	SD	Median	Min	Max
Name agreement	66.25	22.02	68.75	15.62	100
*H*-statistic	1.38	0.82	1.27	0.00	3.66
Normalised *H-*statistic	0.56	0.23	0.61	0.00	0.98
Object recognition RT	1055	222	1015	676	2403
Modal name production frequency	21.32	7.25	22	3	33
Modal name word frequency	4.09	0.85	4.06	1.00	5.44
Modal name word length	5.72	2.29	5.00	2.00	12.00
Weighted average word frequency	4.05	0.64	4.03	1.83	5.44
Weighted average word length	5.79	1.79	5.60	3.00	12.27

#### Within-object agreement

We ran a correlation analysis on the normalised *H*-statistic for all images of the same object to establish the level of within-object naming agreement. Correlation between the minimum and maximum value of *H* associated with each object was strong (*r* = .85), indicating that naming uncertainty varied at the object rather than the image level (i.e. objects tended to attract a similar distribution of unique object names across all four images). Furthermore, 154 objects (77.00% of all 200 objects) had one identical modal name for all four images (average number of unique modal names per object = 1.26, *SD* = .52). Taken together, this suggests naming uncertainty was consistent across multiple images of the same object.

#### Natural versus artefact objects

Objects can be either natural kinds (i.e. occurring in the natural world, such as animals or plants) or artefact kinds (i.e. manufactured by humans, such as tools or vehicles), and previous work has generally found mixed effects regarding differences in processing between these kinds. Some reports suggest that in both normal and patient populations, performance (e.g. RT, accuracy) in object recognition, naming and categorisation is typically better for artefacts than for natural kinds (Humphreys et al., 1988; Warrington & Shallice, 1984). However, these accounts are nuanced by other work, which has shown an advantage for, in particular, animal over artefact categorisation (Proverbio et al., 2007), especially when the objects in question are non-manipulable (i.e. advantage for animals over vehicles: Filliter et al., 2005; McMullen & Purdy, 2006). We therefore report variable characteristics separately for natural and artefact objects.

**Fig. 4:**
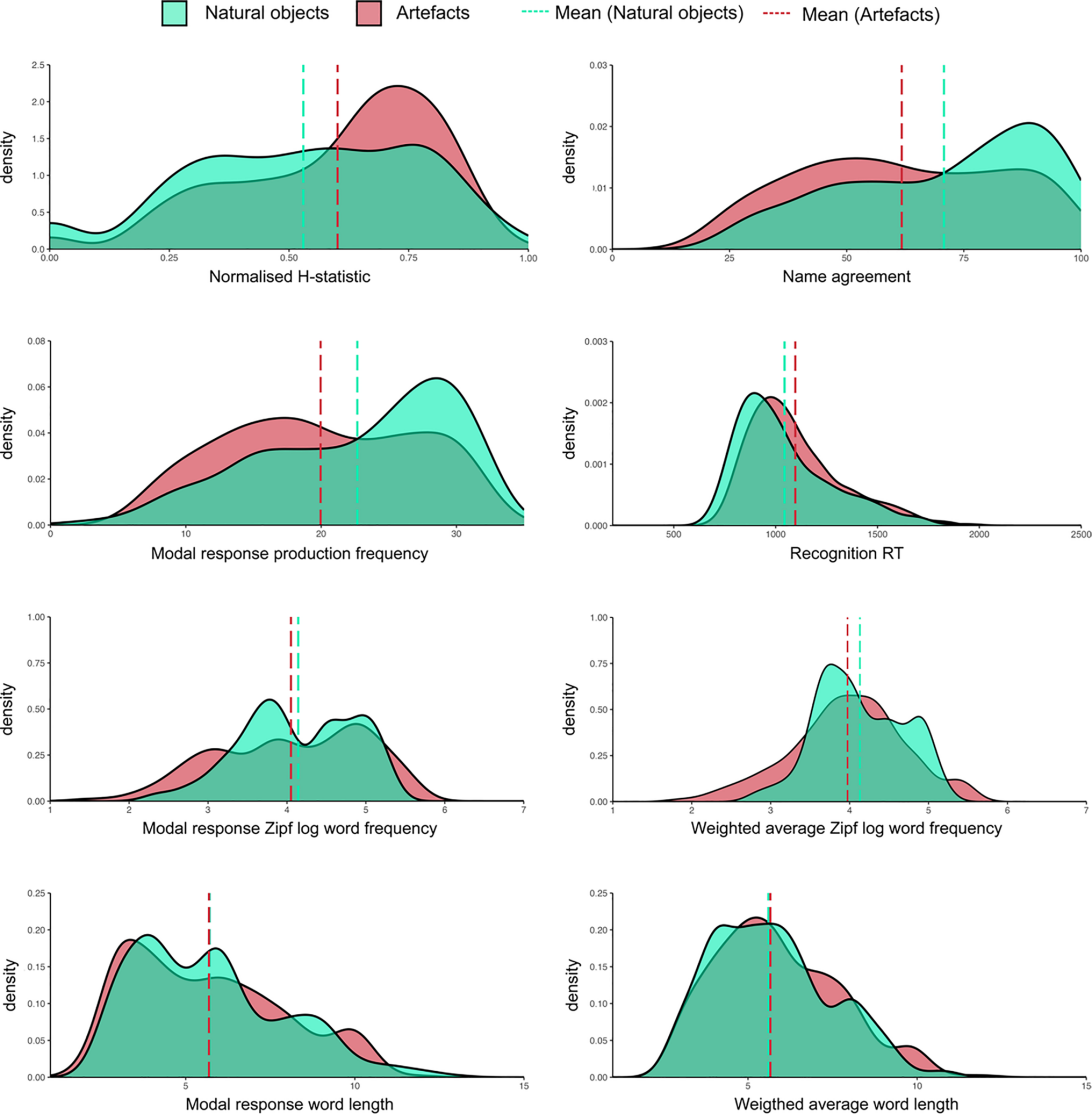
Density plots showing the distributions of modal name production frequency, recognition RT, name agreement, *H*-statistic, weighted average word frequency and length, for natural and artefact objects

Half the objects in the present norms were natural kinds, whereas the other half were manufactured artefacts, and the distributions of many normed variables differed between object type (see Fig. 4). We explored whether these differences were reliable using Welch’s *t* tests (see Table 2). Uncertainty (*H-*statistic) was higher for artefacts than for natural objects. Consequently, name agreement was lower for artefacts, and fewer participants gave the modal name (i.e. lower production frequency) in response to artefacts compared to natural objects. Modal production frequency and weighted average word frequency were both lower for artefacts compared to natural objects, but other lexical characteristics (modal word frequency and length, weighted average word length) did not significantly differ between object types (see also Fig. 4). Overall, recognition RT was faster for natural objects compared to artefacts, which is consistent with some of the existing literature that has demonstrated a processing advantage for natural kinds (e.g. Filliter et al., 2005; McMullen & Purdy, 2006; Proverbio et al., 2007).

**Table 2:** Two-tailed Welch’s *t* tests for the difference in normalised *H*-statistic, name agreement, recognition RT and modal and weighted average word frequency and length between natural objects and artefacts

	Artefacts		Natural kinds			
Variable	*Mean*	*SD*	*Mean*	*SD*	*t*	p*
Name agreement	61.74	21.89	70.75	21.24	– 5.91	< .001
Normalised *H*-statistic	0.60	0.21	0.53	0.24	4.47	< .001
Object recognition RT	1096.42	226.94	1043.52	237.07	3.22	.001
Modal name production frequency	19.95	7.10	22.67	7.18	– 5.37	< .001
Modal name word frequency	4.05	0.95	4.14	0.71	– 1.59	.112
Weighted average word frequency	3.97	0.71	4.13	0.55	– 3.48	< .001
Modal name word length	5.68	2.38	5.70	2.20	– 0.11	0.914
Weighted average word length	5.87	1.82	5.70	1.75	1.35	0.180

*t tests were carried out against a Bonferroni-adjusted alpha level of .006 (.05/8)

## Comparison with previous norms

We chose to compare our study to seven recent picture-naming norms. Of these norms, five used photographic stimuli (Adlington et al., 2009; Brodeur et al., 2010, 2014; Krautz & Keuleers, 2022; Moreno-Martínez & Montoro, 2012; Navarrete et al., 2019), and two used line drawings (Bates et al., 2003; Torrance et al., 2018). Of the studies using photographic stimuli, two were English-language norms, and three were other languages (German, Italian, Spanish). Both studies using line-drawings were multi-language projects, however for the sake of comparison we only report values for their English-language components here.

Other variables recorded in the present study have no counterpart in previous norms, and thus cannot be compared. Word frequency of the modal (or non-modal) names is not consistently included in previous norms, and even where it is, the methodologies for retrieving frequencies have varied. For example, Adlington et al. (2009) and Moreno-Martínez and Montoro (2012) used the log-transformed number of hits for their names (in English and Spanish, respectively) in a popular search engine as their estimate, whereas and Navarrete et al. (2019) report a traditional corpus-derived frequency on the natural log scale. By contrast, in the present work, we used Zipf word frequencies, derived from a large, dialect-appropriate corpus (i.e. British English: van Heuven et al., 2014). Although word length of the modal name can be easily extracted post hoc, the present study also reports word length of the competing non-modal names, as well as the average length weighted over all responses per image, which are not consistently reported in other studies. Furthermore, in contrast to other studies which measured RT from voice recordings Bates et al., 2003) or a combination of first keypress and interkey intervals (e.g. Torrance et al., 2018), the present study recorded RT from the moment a picture appeared on screen to the moment they pressed a key to indicate a name for the object had come to mind (recognition RT).

We therefore focus on overall naming agreement and the response patterns for overlapping items (i.e. objects featured in multiple norms, albeit with different pictures) which can be meaningfully compared across norms.

### Naming agreement

Table 3 shows naming agreement for the present norms compared to previous norming studies. The picture-naming agreement in the present norms is generally on par with existing photographic picture-naming norms with regards to percent agreement and the *H-*statistic. At 66.25% name agreement, a *H-*statistic of 1.38 as well as a mean normalised *H* of 0.56, (*SD* = .23), the present study falls in between previous norms using photographs that reported worse (Brodeur et al., 2010, 2014; Krautz & Keuleers, 2022; Navarrete et al., 2019) or better (Adlington et al., 2009; Moreno-Martínez & Montoro, 2012) levels of agreement. As with most photographic norms, the present study had generally worse naming agreement than those using line drawings as stimuli (Torrance et al., 2018; Bates et al., 2003).

**Table 3:** Comparison of summary statistics with other norming studies

	*N*	Name agreement	*H-*statistic	Language	Stimulus type
Present study	800^a^	66.25 (22.02)	1.38 (0.82)	English (UK)	Photographs
Adlington et al., (2009)	147	67.61 (26.99)	1.11 (0.89)	English (UK)	Photographs
Brodeur et al. (2010, 2014)	1469	59.00 (25.00)	1.86 (1.08)	English (US)	Photographs
Moreno-Martínez and Montoro (2012)	360	72.00 (28.00)	0.94 (0.87)	Spanish	Photographs
Navarrete et al. (2019)	357	56.20 (35.45)	1.49 (1.01)	Italian	Photographs
Krautz and Keuleers (2022)	1547	79.00 (23.00)^b^	0.69 (0.70)^b^	German	Photographs
Torrance et al. (2018)	260	85.00 (19.00)	0.66 (0.75)^c^	English (UK)^c^	Line drawings
Bates et al. (2003)	520	85.00 (16.40)	0.67 (0.61)	English (US)^c^	Line drawings

Table reports average values and standard deviations (in parentheses)

^a^200 unique objects, four images each

^b^Krautz and Keuleers (2022) report name agreement and *normalised* H-statistic for a truncated dataset

^c^Torrance et al. (2018) report median *H-*statistic rather than mean

^d^Results from English language components in multi-language picture-naming studies

### Overlapping items

We compared *H-*statistics for overlapping objects between the present norms and two English-language norms that use photographic stimuli: the British English Hatfield Image Test (HIT, *n*_*overlap*_ = 23; Adlington et al., 2009) and the Canadian English Bank Of Standardised Stimuli sets (BOSS, n_*overlap*_ = 96; Brodeur et al., 2010, 2014).

**Fig. 5:**
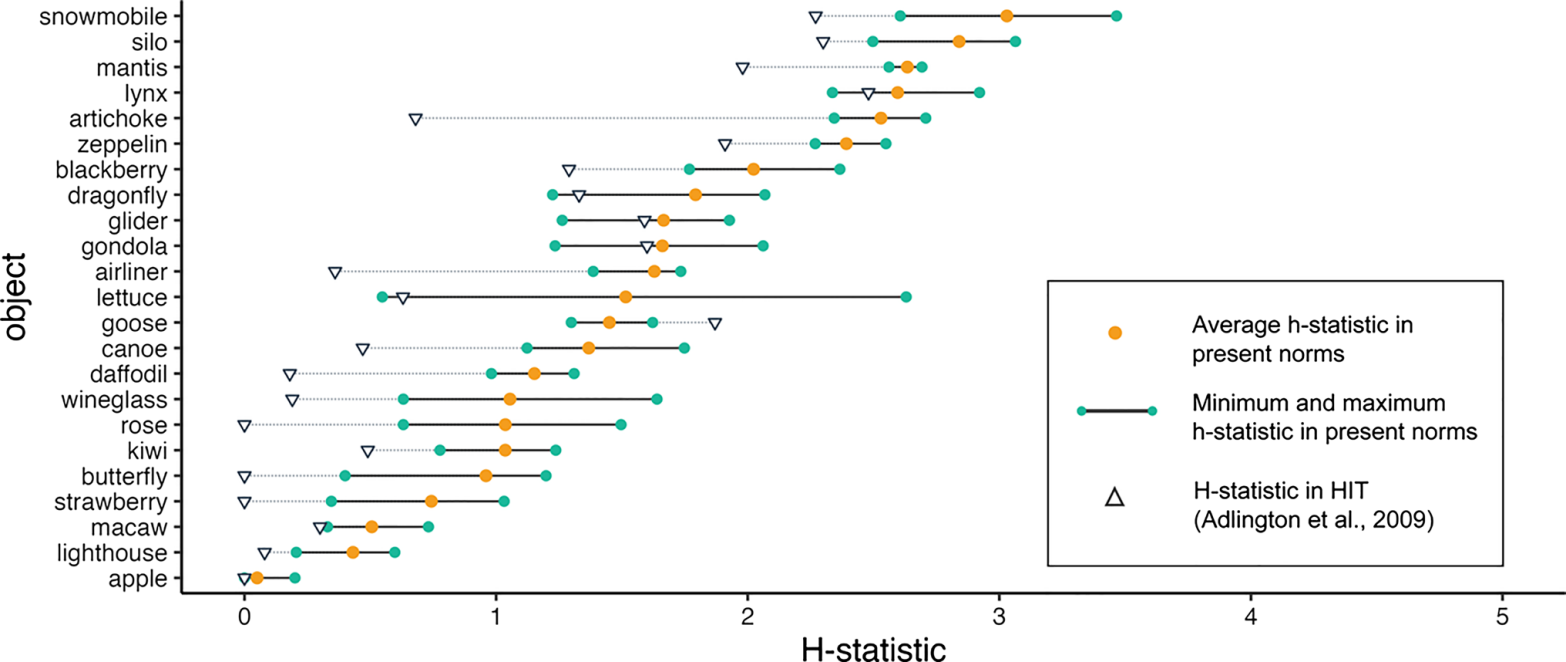
Average, minimum, and maximum *H*-statistic in the present study (across four images per object) compared to the *H*-statistic reported in HIT (Adlington et al., 2009), for 23 overlapping objects. Lower *H*-statistics indicate higher name agreement

Compared to Adlington et al.’s (2009) HIT norms (*M* = .96, *SD* =* .*87; see Fig. 5), the average *H-*statistic for the 23 overlapping objects was higher in the present study (*M* = 1.57, *SD* = .81; *t*(22) = 6.063, *p* < .001), indicating greater diversity in naming. Only one object (*goose*) was named more consistently in the present norms than in HIT; the majority of overlapping objects had the opposite pattern. The greatest level of divergence was for the object *artichoke*, which received the modal name of *artichoke* in both norms but with a high level of consensus amongst HIT participants (*H-*statistic = 0.68) compared to low consensus amongst participants of the present norms (average *H-*statistic = 2.53) with a diverse range of alternative names (i.e. *vegetable, plant, flower, bulb, fruit, broccoli, plant, cactus, bud, shrub, asparagus, food, coral, avocado, sprout and flower bud).* Overall, however, the *H-*statistics (averaged over all four images associated with each object in the present norms) correlated strongly between norms: *r*(21) = .84, *p < .*001.

Nonetheless, even where the degree of naming diversity was similar, the relative frequency of names often differed between the present norms and the HIT. For example, while the average *H-*statistic for images of gondola (*H-*statistic = 1.66) in the present norms was similar to that of the HIT (*H-statistic* = 1.60), the frequency distribution of responses was different: *boat* was the modal response for three out of four gondola images in the present norms, with *gondola* the modal response for the remaining image and a frequent alternative to the others. By contrast, the HIT recorded the reverse (i.e. *gondola* as the most frequent response, with *boat* the most frequent alternative). Furthermore, in some cases, the images in the present norms were given names that did not occur in the HIT and vice versa. For example, participants in the present norms agreed with HIT participants that a dragonfly is most frequently named *dragonfly* but diverged in the alternative names: our participants noted that it can also be called a generic *insect*, *bug*, or *fly*, *horse fly* and even *grasshopper* whereas HIT participants opted for the more specific names of *mosquito, lacewing*, *mayfly,* and *moth*.

**Fig. 6:**
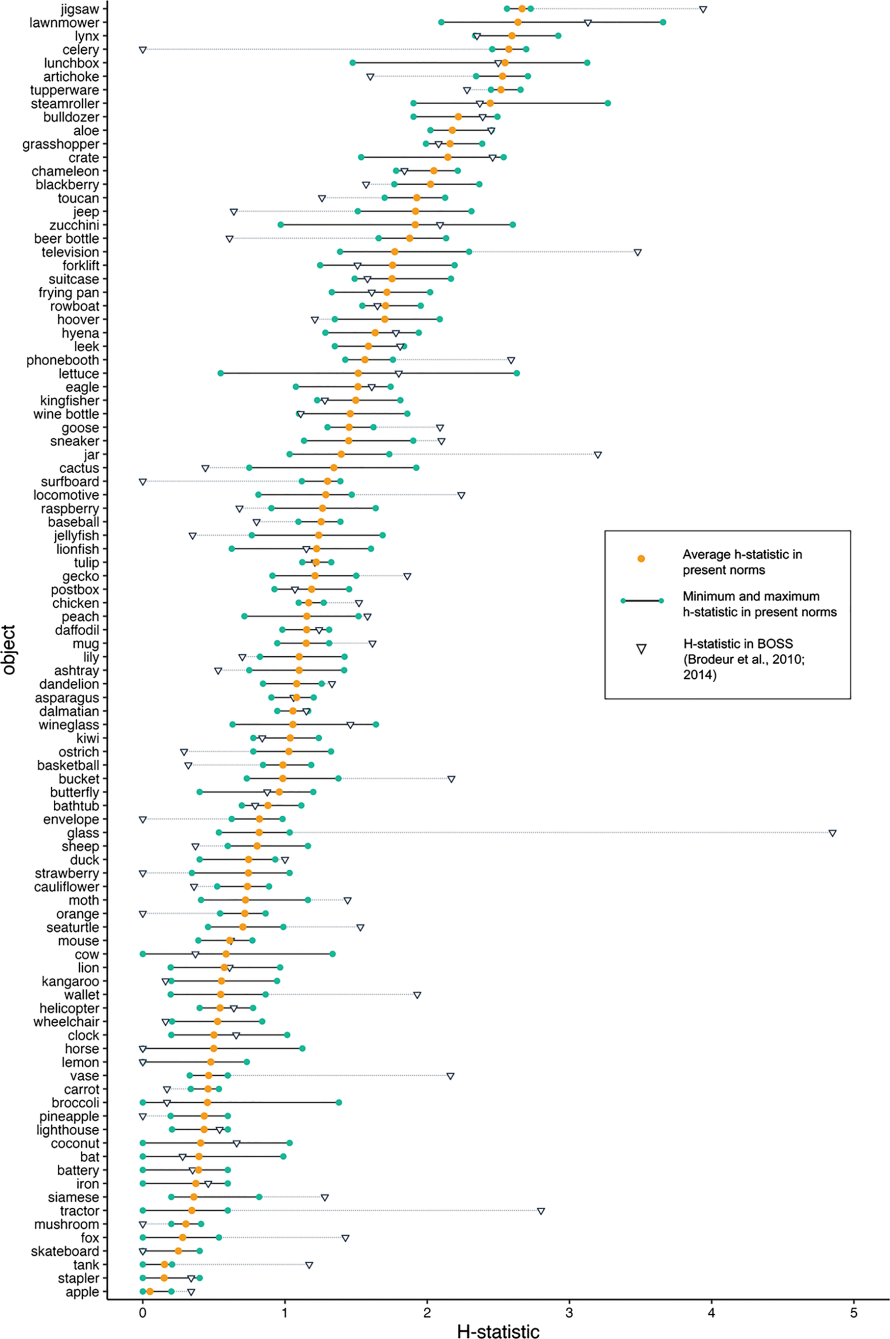
Average, minimum, and maximum *H*-statistic in the present study (across four images per object) compared to the *H*-statistic reported in the BOSS picture set (Brodeur et al. 2010, 2014), for 96 overlapping objects. Lower *H*-statistics indicate higher name agreement

Compared to Brodeur et al.’s BOSS norms (*M* = 1.25, *SD* = .97; see Fig. 6), the average *H-*statistic for 96 overlapping objects did not differ significantly in the present study (*M* = 1.18, *SD* = .68; *t*(95) = – 0.770, *p* = .443). We found that the average *H-*statistic per overlapping object in the present study correlated moderately with that reported in the BOSS set, *r*(94) =.54 , *p* = < .001.

Since the BOSS norms contain only modal and not alternative names, we could not compare the relative frequency of all names produced per object. Nonetheless, some objects were named in a very similar way across both sets of norms, such as kiwi, which had the modal name *kiwi* in the present norms (also called *fruit, kiwifruit* and *coconut*) and modal name *kiwi* in the BOSS norms, with relatively similar *H*-statistics (1.03 and 0.84, respectively). Other objects, however, were markedly different in their naming patterns. While BOSS participants all agreed that an image of celery should be called *celery* (*H-*statistic = 0.00), only just over a third of participants in the present norms settled on the modal name *celery* (average *H-*statistic = 2.57), with the others producing alternative names including *vegetable, herb, parsley*, *leek, leaf, coriander, onions, greens, chives, food, salad* and *plant*. By contrast, the greatest positive divergence was for the object glass, which was named relatively consistently in the present norms (modal name *glass*, average *H-*statistic = 0.81) but had a high degree of uncertainty in the BOSS norms (only a minority of 27.03% used the modal name *glass*: *H-*statistic = 4.85).

There are a number of possible explanations for the differences in naming diversity between norms. Firstly, the choice of image may have influenced participants’ familiarity with the object and therefore their choice of name. For example, in the case of celery, all of the images in the present norms depicted the vegetable in an upright position, with clearly visible leaves. By contrast, the celery image in the BOSS norms (Brodeur et al., 2010, 2014), depicted a horizontally positioned vegetable without visible leaves. While both depictions are valid, it is possible that greater familiarity with leafless celery may have led to its lower naming uncertainty in the BOSS norms. Conversely, images in the present norms consistently depict a glass as a colourless, transparent, drinking glass, whereas the glass object used in the BOSS norms was a blue and opaque cup-shaped object. Here too, greater familiarity with transparent, colourless glassware may have led to fewer competing names (e.g. *cup*, *container*, *tumbler*, *water*) in the present norms. Secondly, there may be an effect of cultural and dialectal differences. As outlined above, the degree of naming consensus amongst British participants in the present norms correlated more strongly with the UK HIT norms (Adlington et al., 2009) than the Canadian BOSS norms. A baseball, for instance, attracts more diverse names in the present norms (*baseball*, *ball, cricket ball*) than in the BOSS norms, which is consistent with the lower popularity of baseball as a sport in the UK. Nonetheless, it is worth noting that even where dialectal differences exist, it may not affect naming diversity: the *H*-statistics for the zucchini object are similar in the present and BOSS norms even though the modal name itself differs (i.e. a *zucchini* in Canada is a *courgette* in the UK).

## Predicting recognition latencies

The goal of this final analysis was to validate the latencies recorded in the present norms, and additionally to determine whether our new measures of weighted word frequency and length – reflecting the diversity of names produced for each picture – could predict these latencies better than such variables based on the modal name only.

In the present norms, we recorded the time it took for participants to indicate that a name for the depicted object had come to mind (recognition RT). While this measure differed from previous work which relied on voice recordings (Bates et al., 2003; Johnston & Barry, 2006; Snodgrass & Yuditsky, 1996), it is closer in nature to the first-keypress measure used in Torrance et al. (2018). Torrance and colleagues found that first-keypress RT decreased as *H*-statistic decreased (i.e. greater uncertainty leading to slower responses), mirroring the pattern previously found for voice recordings (e.g. Barry et al., 1997; Székely et al., 2003). To validate the RT measure in the present norms, we therefore expected the same pattern to appear between uncertainty and latency, with a higher *H-*statistic resulting in slower recognition RT.

In addition, previous work has found variable evidence for the effects of word frequency and length on picture-naming (Barry et al., 1997; Johnston & Barry, 2006; Perret & Bonin, 2019). Some of this variability may be explained by the variability in the sources and measures used to determine word frequency, which may affect its efficacy as a predictor of RT (e.g. van Heuven et al., 2014). However, it may also be the case that the usual practice of predicting picture-naming latencies through psycholinguistic properties of *only* the modal name can be improved by using weighted measures of all names produced in response to a given image. That is, since few pictures are named with a single label by all participants, and since the modal name constitutes a minority of responses for many objects with moderate or high uncertainty, it seems sensible to take into account the diversity of names that are used to label an image, weighted by the frequency with which they are produced.

We therefore tested whether recognition RT was better predicted by the *weighted average* word frequency and length of all non-idiosyncratic names per image than the usual *modal* word frequency and length of the modal name alone. By incorporating relative production frequencies, these weighted frequency and length variables reflect the probability of name selection across a group of participants.^5^ We expected this extra information to enhance the ability of word frequency and length to explain picture recognition latencies.

### Method

#### Materials

For this analysis, we used the image-level norms of 800 items that included mean recognition RT for every image (i.e. averaged over all responses after the removal of invalid, unknown and outlier responses). Across all images, we collated *H-*statistic (*M* = 1.38, *SD* = .82), modal word frequency (*M* = 4.10, *SD* = .84), modal word length (*M* = 5.70, *SD* = 2.29), weighted average word frequency (*M* = 4.05, *SD* = .64), and weighted average word length (*M* = 5.79, *SD* =1.79) per image.

#### Analyses

First, to determine whether decreasing *H-*statistic reduced recognition RT and thereby validate our dependent variable, we ran a linear regression on recognition RT with *H-*statistic as the independent variable. We report Bayes factors (BF_10_) for model fit against the null (empty) model, as well as frequentist statistics for model fit and coefficients.

Second, to determine whether weighted-average word frequency and length were better predictors of recognition RT than modal word frequency and length, we ran two sets of separate hierarchical linear regression models with recognition RT as the dependent variable and compared their respective performance. The first regression (modal-name model) added independent variables of modal word frequency at Step 1 and modal word length at Step 2. In the second regression (weighted average model), we added independent variables of weighted word frequency at Step 1 and weighted word length at Step 2. For both models, we report Bayes factors (BF_10_) for each successive step, as well as frequentist statistics for model fit and coefficients at Step 2. used Bayesian model comparisons with Bayes factors (BF_10_) calculated from BIC (see Wagenmakers, 2007) to test model fit at each step. Finally, by taking the Bayes factors of the Step 2 models, we were able to use non-nested Bayesian model comparisons to determine whether the RT data were best fit by the modal-name model (H_0_) or the weighted-average model (H_1_).

### Results and discussion

#### *H*-statistic

Recognition RT was successfully predicted by *H-*statistic, with a very strong level of Bayesian evidence (BF_10_ = 10.71 × 10^93^) and an adjusted *R*^*2*^ of .423, *F*(1, 798) = 587.24, *p* < .001. As expected, participants were up to 678.07 ms slower to respond to images with the highest naming uncertainty (3.66) compared to the lowest (0.00); unstandardised *b* = 185.27, *SE* = 7.64, *t* = 24.23, *p* < .001. That is, as with latencies in vocal naming and first-keypress latencies, the latencies for recognition keypress in the present naming norms increased with naming uncertainty.

#### Modal versus weighted-average word frequency and length

In the modal-name model, Bayesian model comparisons very strongly favoured modal word frequency as a predictor of recognition RT at Step 1 (BF_10_ = 8.36 × 10^13^), but was equivocal about the inclusion of modal word length at Step 2 (BF_10_ = 0.50). The Step 2 modal-name model was still better than the null (BF_10_ = 4.16 × 10^13^) and explained recognition RT at adjusted *R*^*2*^ = .088, *F*(2, 797) = 38.47, *p* < .001; see Table 4 for coefficients. Participants were faster to respond to images with higher-frequency modal names (i.e. 93 ms faster for each Zipf-unit of increase), but – counterintuitively – modal word length *decreased* RT (i.e. 8 ms faster for each extra letter in the name). However, since zero-order correlations showed a *positive* relationship between RT and modal word length (*r* = .149), that was weaker than the relationship between modal word length and frequency (*r* = – .673), we concluded the negative regression coefficient for modal word length was a suppression artefact (e.g. Friedman & Wall, 2005) of modal word frequency.

**Table 4:** Linear regression coefficients of recognition RT for full modal-name and weighted-average models

Predictor(s)	Unstandardized *b*	95% CI	*t*	*P*
Modal-name model				
Modal word frequency	– 96.38	± 24.96	– 7.58	< .001
Modal word length	– 8.55	± 9.14	– 1.84	.067
Weighted-average model				
Weighted word frequency	– 145.24	± 31.86	– 8.96	< .001
Weighted word length	– 18.45	± 11.42	– 3.12	.002

In the weighted-average model, by contrast, Bayesian model comparisons favoured adding *both* variables, with very strong evidence for weighted average word frequency at Step 1 (BF_10_ = 4.74 × 10^15^) *and* evidence for weighted average word length at Step 2 (BF_10_ = 12.66). The Step 2 weighted-average model of recognition RT was much better than the null model (BF_10_ = 6.00 × 10^16^), with an adjusted *R*^*2*^ = .103, *F*(2, 797) = 46.65, *p* < .001. Participants were again faster to respond to images when the weighted-average frequency across all names was higher (i.e. 145 ms faster for each Zipf-unit of increase). As with modal word length, the Step 2 model suggested that weighted-average word length decreased RT (i.e. 18 ms faster for each extra letter); however, since zero-order correlations again showed that weighted word length was *positively* related to RT (*r* = .122) and strongly related to weighted word frequency (*r* = – .660), we again concluded its negative coefficient was a suppression artefact.^6^

Critically, Bayesian model comparisons showed that the weighted-average model was BF_10_ = 1442.50 times better than the modal-name model in fitting the data. That is, word frequency and length of the modal (i.e. most common) name did predict how quickly an object name came to mind in a picture-naming task, but not as well as the weighted-average word frequency and length of *all* non-idiosyncratic names given to an image. This finding suggests that the weighted variables provided in our norms may be more useful to researchers than merely focusing on the modal name alone.

## Conclusions

The timed picture-naming norms we present here differ from extant norms in a number of ways. In contrast to other influential norms such as the Snodgrass and Vanderwart set, as well as the extended set presented by Bates et al. (2003), the present norms contain high-resolution photographs rather than line drawings. Furthermore, in contrast to other norms which have used photographic stimuli the present set systematically incorporates multiple images of the same object, each with its own normed variables, in order to enable greater flexibility in stimulus selection and experimental design.

Finally, the present norms contain measures of word frequency and length not only for the modal (i.e. most common) name of each picture, but also for all names given to a picture as a weighted average of how often each name was produced. Our analysis shows that these weighted-average variables outperform modal-name variables in predicting RT, meaning that that *all* likely names of an object – and not only the most common one – affect the speed with which participants process its image. While researchers interested in object recognition and naming could simply restrict their item selection to pictured objects with minimal uncertainty in their naming (e.g. where > 90% of participants agree on a particular modal name), such an action would produce unrepresentative stimulus sets whose results may not necessarily generalize to object processing as a whole. Most objects have multiple possible labels, and so such diversity of naming behaviour should be incorporated in experimental designs (e.g. by using relevant weighted-average variables as predictors or baseline controls).

We hope the norms presented here are a useful resource for researchers interested in any aspect of object recognition and naming and will allow researchers more choice and control over the selection of their stimuli.

## Footnotes

1 Testing in this way meant it was possible for participants to see and name all four images associated with a single object (e.g., four images of *poodle*). However, across 60 participants and 200 objects with four images each, the median number of responses per object coming from a single participant was two.

2 We defined the modal response as that having the highest production frequency per image. For 17 images, maximum production frequency was tied between two responses. For these images, we opted to select the response that most closely matched the majority of modal names for the other images of the same object. For example, the most frequent names for the poodle_2 image were *poodle* and *dog*. Since the majority modal response for the other three poodle images was *poodle*, we opted to break the tie for poodle image 2 in favour of *poodle*.

3 In the trial-level data (see supplemental materials on OSF), we report both recognition RT per trial and a measurement of the time to initiate typing from presentation of the text box (first-key RT); as a paradigm check, 99.98% of valid typed responses were initiated within the motor planning window of 300 ms (M = 93 ms, *SD* = 31 ms).

4 For example, the Canoe_3 image was named as canoe, boat and kayak, with respective word frequencies of 3.50, 4.89 and 3.27, and production frequencies of 19, 11 and 1 out of 31 participants who saw the image. We therefore excluded *kayak* as a response (production frequency was only 1) and calculated weighted word frequency = (19*3.50 + 11*4.89) / 30 = 4.01.

5 We are agnostic as to whether the weighted variables indirectly reflect the probability of selecting a given object name *within each participant* (i.e. lexical competition), although recent work suggests it may be partially the case (Balatsou et al., 2022).

6 We ran a linear mixed model of trial-level RT (see supplemental materials), with word frequency and length of the actual response provided by the participant in each trial; results were similar to the image-level analysis, with a strong effect of word frequency and a weak effect of word length without the suppression artefact (i.e. slower RT for object names with lower lexical frequency and longer word length).
